# Differential Fecal Microbiome Dysbiosis after Equivalent Traumatic Brain Injury in Aged Versus Young Adult Mice

**DOI:** 10.33696/neurol.2.044

**Published:** 2021

**Authors:** Booker T Davis, Mecca B.A.R. Islam, Promi Das, Jack A Gilbert, Karen J. Ho, Steven J. Schwulst

**Affiliations:** 1 Department of Surgery, Division of Trauma and Critical Care; Northwestern University, Chicago Il, USA; 2 Department of Pediatrics and Scripps Institution of Oceanography, University of California San Diego, La Jolla, CA, USA; 3 Department of Surgery, Division of Vascular Surgery, Northwestern University, Chicago Il, USA

**Keywords:** Traumatic Brain Injury, Age, Microbiome, Dysbiosis, Trauma, Controlled Cortical Impact

## Abstract

Traumatic brain injury (TBI) has a bimodal age distribution with peak incidence at age 24 and age 65 with worse outcomes developing in aged populations. Few studies have specifically addressed age at the time of injury as an independent biologic variable in TBI-associated secondary pathology. Within the framework of our published work, identifying age related effects of TBI on neuropathology, cognition, memory and motor function we analyzed fecal pellets collected from young and aged TBI animals to assess for age-induced effects in TBI induced dysbiosis. In this follow up, work we hypothesized increased dysbiosis after TBI in aged (80-week-old, N=10) versus young (14-week-old, N=10) mice. C57BL/6 males received a sham incision or TBI via open-head controlled cortical impact. Fresh stool pellets were collected 1-day pre-TBI, then 1, 7, and 28-days post-TBI for 16S rRNA gene sequencing and taxonomic analysis. Data revealed an age induced increase in disease associated microbial species which were exacerbated by injury. Consistent with our hypothesis, aged mice demonstrated a high number of disease associated changes to the gut microbiome pre- and post-injury. Our data suggest divergent microbiome phenotypes in injury between young and aged reflecting a previously unknown interaction between age, TBI, and the gut-brain axis implying the need for different treatment strategies.

## Introduction

Traumatic brain injury (TBI) is defined as an external mechanical force that leads to an acquired brain insult. TBI can be derived from a range of principal mechanisms including falls, assaults, self-harm, and motor-vehicle crashes. Notably, specific causes of TBI correlate with age. Motor vehicle crashes are greatly associated with age groups 15-24 years old, while falls are a leading cause of TBI amongst adults aged 65 years and older [1]. Each year in the United States, there are nearly 3 million TBI-related emergency department visits, hospitalizations, and deaths resulting in 80-90,000 chronic or permanent disabilities [2,3]. As such, brain injury is a serious health concern, yet no viable therapeutics other than supportive care exist to help the underlying deleterious processes that are initiated by the initial injury [4,5].

Regardless of injury mechanism, TBI is a heterogenous injury process that encompasses mechanical tissue disruption, neuronal excitotoxicity, free radical generation, disruption in energy metabolism, and neuroinflammation [6,7]. All of these processes have the potential to culminate in a spectrum of motor, cognitive, and behavioral disability [8]. However, it is clear that the pathophysiology of injury varies between the developing and mature brain [9]. The young brain is highly active in the processes of development and growth, resulting in different patterns of injury, repair, and regeneration as compared to the aged brain [10]. In fact, the etiology and evolution of TBI symptoms in young patients is well documented in the literature demonstrating patterns of impairment in communication, behavior, higher-order cognition, and learning efficiency [10-12].

On the other hand, TBI in aged populations has not been as well studied. The best available evidence shows patterns of reduced communication skills, memory-compensation strategies, and greater overall loss of physical and cognitive function in the aged TBI population [1,13]. Some have speculated that this is due to a diminished repair response in the aged patient as well as the presence of age-related neurodegenerative changes that are accelerated and exacerbated at the time of injury [14,15]. For example, young patients with brain injuries demonstrate improved rates of recovery and better functional outcomes when compared to their aged counterparts [16]. Our recently published findings *“Differential Neuropathology and Functional Outcome After Equivalent Traumatic Brain Injury in Aged Versus Young Adult Mice”* we used MRI, histological, and behavioral tests to investigate aged-induced effects on the secondary pathology associated with TBI [17]. Our analysis revealed an unexpected age-based attenuation of white matter connectivity and neuropathology. More specifically, we found that aged mice (80 weeks old) demonstrated less cerebral edema and attenuated neuronal loss within the cortex and subcortical grey matter as compared to young-adult mice (14 weeks old). Hippocampal gliosis was severe in both groups. One the other hand, young-adult mice demonstrated severe and extensive edema, neuron loss, and gliosis within the cortex, hippocampus, and subcortical grey matter as compared to aged mice. Lastly, we observed significant age effects on anxiety, memory and learning between young adult and aged adult male mice following an identical impact injury. In the current study, we examined fecal samples collected from the mice studied in the aforementioned work. We believe that this comprehensive assessment is a necessary addition to the literature and provides a better global understanding of age-effects on TBI outcomes [17].

An understudied domain that could be contributing to this age-related differential in TBI outcome is the gut microbiome. The gut microbiome is known to change with age and is implicated in a myriad of physiological processes including health, inflammation, and development in young and aged populations [18,19]. Brain injury is known to disrupt the bidirectional communication between the central and the enteric nervous systems known as the brain-gut axis (BGA) [20-23]. It is thought that TBI results in activation of the sympathetic, parasympathetic, and hypothalamic-pituitary-adrenal axis, resulting in dysbiosis and disease through a number of yet-to-be discovered mechanisms [24,25]. In fact, TBI-induced alterations in the gut microbiome have been documented as early 2 hours postinjury. Furthermore, microbial divergences have been correlated to the development of neurologic and systemic diseases ranging from inflammatory bowel disease to Alzheimer’s disease [22,25-27].

Prior studies provide evidence of TBI-directed loss of beneficial gut bacteria postinjury [20-23]. However, the data are limited to acute and extreme chronic time points while intermediary processes remain undefined. We, therefore, analyzed fecal pellets collected during our aforementioned age TBI mouse study, with the aim of further illuminating the breadth of age-based effects in TBI. We evaluated whether the aged gut microbiome could impact TBI during recovery period. We hypothesized that aged mice would demonstrate increased gut dysbiosis after TBI as compared to young mice.

## Methods

### Animals

Twenty C57BL/6 male mice (Mus musculus) (29-31 grams) were purchased from the Jackson Laboratory (Bar Harbor, Maine). Ten of the twenty mice were ordered 60 weeks prior to the start of the experiment and aged to 80 weeks in-house. Young groups were ordered and given 2 weeks of facility acclimation time before the start of the experiment, which was concurrent for all groups. Mice were maintained in a pathogen-free barrier facility at the Northwestern University Center for Comparative Medicine during the study period and at Jackson laboratories prior to arrival. Consistent NIH-31 formulation Chow and water were provided ad libitum at both Jackson Laboratory and Northwestern

University. Animal weights were matched between experimental groups. Bedding transfer and mixing was performed 2 weeks prior to TBI. Mice were treated in accordance with the National Institutes of Health Guidelines for the Use of Laboratory Animals. The experimental protocol was approved by Northwestern University Institutional Animal Care and Use Committee. Results of histological analyses for the animals within the current study were published as a part of the manuscript *“Differential Neuropathology and Functional Outcome After Equivalent Traumatic Brain Injury in Aged Versus Young Adult Mice”* [17].

### Traumatic brain injury

Mice were anesthetized using an intraperitoneal injection of 10 mg/kg xylazine (Anased, Shenandoah, IA) and 125 mg/kg ketamine (Ketaset, Fort Dodge, IA). Following anesthesia, a 1-cm scalp incision revealed the sagittal and coronal sutures of the skull. The injury site is marked 2 mm rostral to the coronal suture and 2 mm left of the sagittal suture. A 5 mm-diameter impact area of the brain is exposed via a craniectomy leaving the dura mater intact. TBI mice were stabilized within a stereotaxic operating frame. A commercially available impacting device (Impact One, Leica Biosystems, Des Planes IL) was utilized to induce a controlled cortical impact. The impacting rod was 3 mm in diameter and deployed at a velocity of 2.5 m/s to an impacting depth of 2 mm with a 0.1 second dwell time. Sham mice underwent anesthesia and scalp incision alone.

The scalp incisions of all groups were sealed with VetBond (3M) (Santa Cruz Animal Health, Dallas, TX) immediately following sham injury or TBI. Post-procedure analgesia with Buprenorphine SR (SR Veterinary Technologies, Windsor, CO) was administered to all animals via subcutaneous injection. Animals were recovered in separate cages over a warming pad. Euthanasia occurred at 30 days post injury via carbon dioxide inhalation, perfusion, and decapitation. Brains were harvested for analysis by immunohistochemistry.

### DNA extraction and sequencing

Mice were housed separately for 2 hours on days of stool collection. Stool samples were flash frozen in liquid nitrogen and stored at −80°C until use. Individual stool pellets were weighed. DNA extraction was performed using the PowerSoil DNA Isolation Kit (Qiagen) according to the manufacturer’s instructions. DNA quantitation was estimated using a spectrophotometer NanoDropR ND-1000 (NanoDrop Technologies, DE, USA). We used a spectrum absorbance/transmission ratio of 260/230, passing light through the DNA in liquid medium to determine the concentration. Samples were then shipped to the Gilbert Laboratory, University of California, San Diego for 16S rRNA processing. Samples were loaded into 96 well plate and using MagAttract Power Microbiome DNA/RNA KF following protocol for DNA extraction [28]. Follow by a 23 μl PCR reaction contained a mixture: 9.5 μl of MoBio PCR Water (Certified DNA-Free; Mo Bio Laboratories), 12.5 μl of 5-Prime HotMasterMix (1×), 1 μl of forward primer (5 μM concentration, 200 pM final), 1 μl of Golay Barcode Tagged Reverse Primer (5 μM concentration, 200 pM final), and 1 μl of template DNA. The conditions for PCR were as follows: 94°C for 3 min to denature the DNA, with 35 cycles at 94°C for 45 s, 50°C for 60 s, and 72°C for 90 s, with a final extension of 10 min at 72°C to ensure complete amplification. Amplicons were quantified using PicoGreen (Invitrogen) assays and SpectraMax iD3 Multi-Mode Microplate Reader, followed by clean up using UltraCleanR PCR Clean-Up Kit (MoBio, Carlsbad, USA) and then quantification using Qubit readings (Invitrogen, Grand Island, USA). DNA was diluted in 100 ul with nuclease-free HyClone Molecular Biology-Grade Water. The V4 region of the 16S rRNA gene (515F-806R) was amplified with region-specific primers that included the Illumina flow cell adapter sequences and a 12-base barcode sequence. The 16S rRNA sequencing on an on the Illumina HiSeq 2000 platform (2 × 150 paired-end sequencing) was conducted at the IGM Genomics Center, University of California, San Diego, La Jolla, CA. according to Earth Microbiome Project [29] standard protocols [28].

### 16S rRNA gene data analysis

QIIME 2 V2019.10 was used to process the reads [30]. The input files used were the paired end reads in fastq format and a mapping file with the barcode sequence corresponding to each sample. Reads were split by sample-specific barcode, followed by denoising using the DADA2 plugin. Taxonomic classification was performed using the naive Bayes pretrained QIIME2 classifier based on the Greengenes reference database 13_8. Samples with low count of reads per sample were excluded and the rest were rarefied to a depth of 3500 sequences per sample. Alpha diversity (Faith’s PD, Shannon diversity, and observed operational taxonomic unit (OTU), richness) for various groups was generated and compared with a Kruskal-Wallis test. For beta diversity, pairwise unweighted and weighted UniFrac distances were generated and then the distances of the between-group differences were tested using PERMANOVA and permuted t tests in QIIME 2. The boxplots and the heatmap were produced using the relative abundances of the microbes at phyla and species level of taxonomic lineage using the package *ggplot2* and *heatmap.plus* in R V3.6.1 respectively.

## Results

### Microbiome analysis

**Figure 1:**
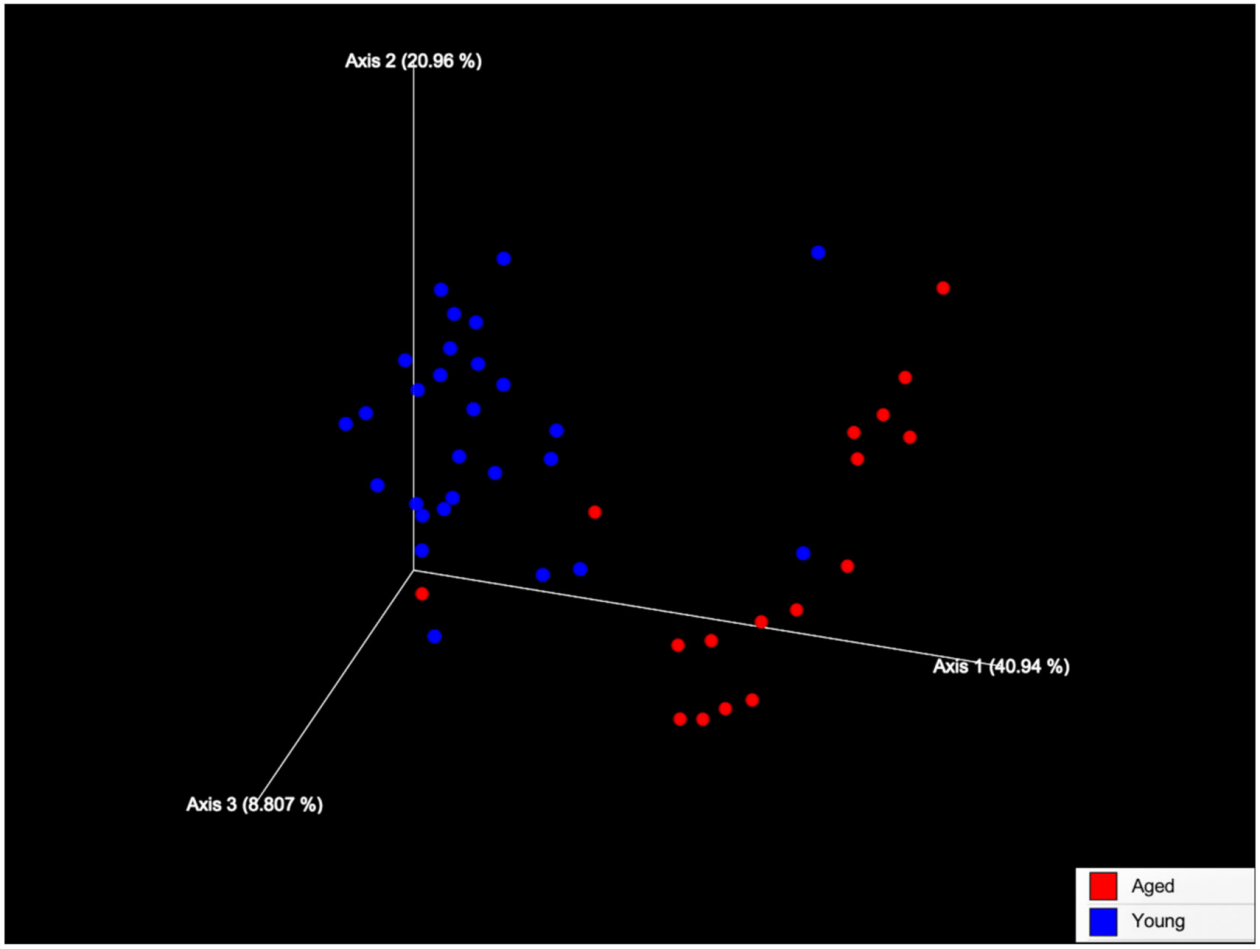
Beta diversity within the fecal bacterial microbiome in young and aged mice. Fecal microbiome beta diversity represented as a Principal Coordinates Analysis (PCoA) plot of all subjects at 1, 7, and 28 days post injury based on 16S rRNA amplicon sequencing (N = 18). Each circle represents an individual mouse. Samples are colored by age: young (Blue) mice and aged (Red) mice. PCoA directionality and association indicates similarity of bacterial profiles within subjects. Permutational Multivariate Analysis of Variance (PERMANOVA), age effect was significant (p<0.001).

We examined age and injury related alterations using 16S analysis of the fecal microbiome. Fresh stool pellets were collected 1-day pre-TBI, then 1, 7, and 28-days post-TBI. The conserved variable region within 16S rRNA genes was then sequenced for identification, classification, and quantification of the various microbes contained within the stool specimens. Certain statistical comparisons could not be made between groups when bacterial reads were lower than our threshold and these data points were excluded; trends are shown. Beta diversity analysis was carried out using Principal Coordinates Analysis (PCoA). Each data point represents the taxonomic assignment of 16S rRNA found in each sample and reveals the difference in relative species abundance (Figure 1). The data revealed striking differences in the distributions of fecal bacteria between young and aged mice *(p<0.001)*. The search for differences between young and aged microbiomes according to time, before and after injury, revealed no significance (Figure S1).

**Figure 2:**
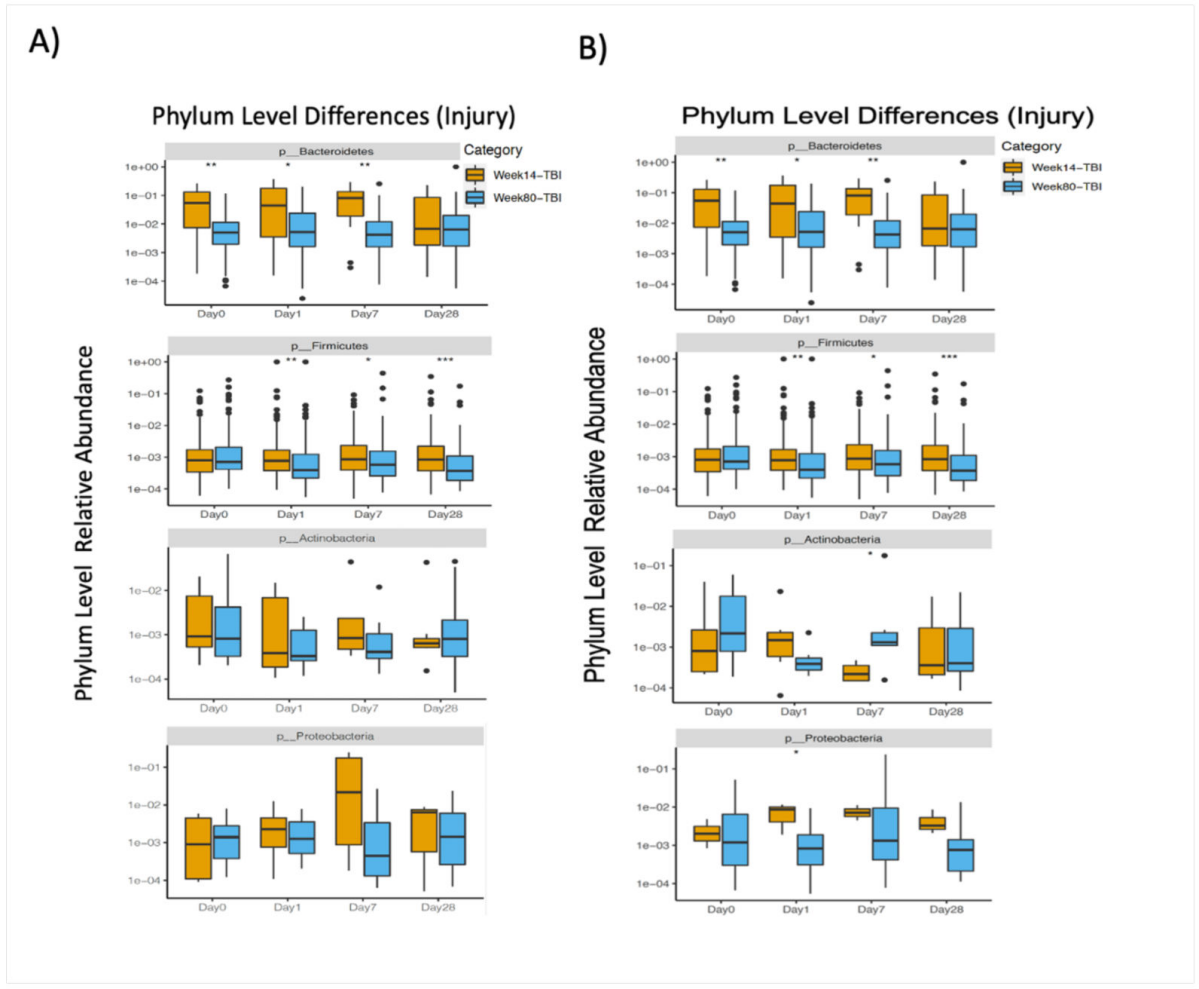
Phylum level alterations in the fecal microbiome of young and aged mice after TBI. Box plots showing the relative abundance of V4 region of 16S rRNA between young and aged mice at baseline and up to 30 days post-TBI. (**A**) Phyla-level analysis demonstrates significant baseline age-related differences in the relative abundance of OTUs in fecal samples between young and aged mice (p ≤ 0.05(*), 0.001(**), & 0.0001(***)). (**B**) At 30 days post-injury, phyla-level differences in the relative abundance of OTUs in fecal samples expanded to include 4 significantly altered phyla after injury ((p ≤ 0.05(*), 0.001(**), & 0.0001(***)).

In order to determine an interaction between age and TBI on gut microbiota, we assessed phyla level differences over the course of injury in both young and aged mice post-TBI. Overall, we found that the phyla level revealed more detailed changes with treatment compared to beta diversity results. Similar to the beta diversity results we found significant age-dependent alterations in the gut microbiome at the phyla level. The increased level of detail at the phyla level also showed evidence of injury-dependent differences that were not discernable in beta diversity (Figure 2A). Baseline (Day 0) analysis revealed significant decrease in Bacteroidetes and Firmicutes in aged mice as compared to young mice (p<0.001). This age-related difference in phyla fluctuated over time following experimental TBI but resurfaced by post-injury day 30. On the other hand, analysis of the TBI groups revealed greater differences in the relative abundance of phyla in both the young and aged groups over the course of injury ((p < 0.05(*), 0.001(**), & 0.0001(***)). While Bacteroidetes was the only significantly different phyla at baseline in these animals, differential changes in Bacteroidetes, Firmicutes, Actinobacteria, and Proteobacteria were seen in TBI animals over the course of the study (Figure 2B).

**Figure 3:**
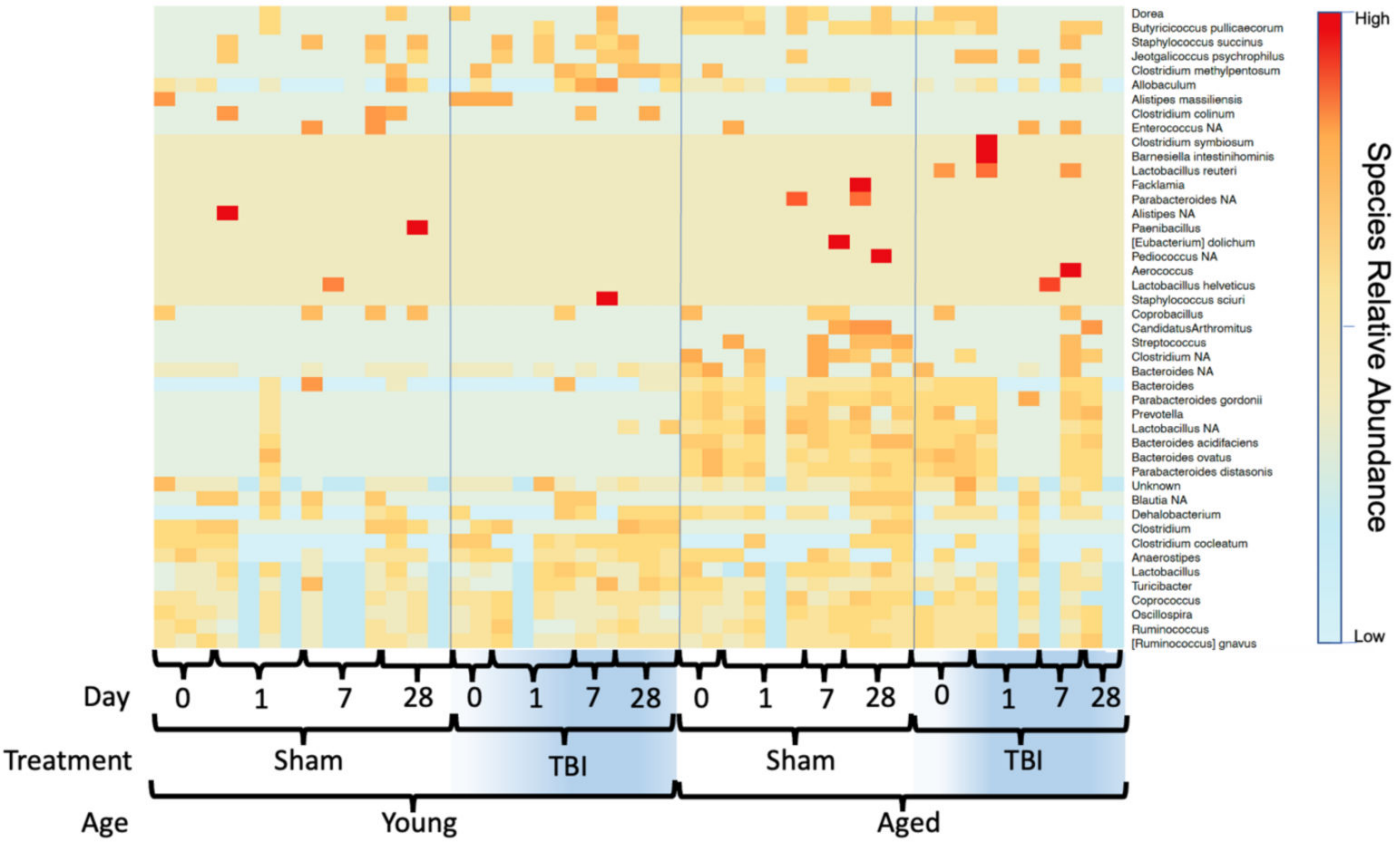
Relative species-level expression in the fecal microbiome of young and aged mice after TBI. A heatmap plot was generated to represent the differences in the microbial community at the species level.

Given both the age and TBI-related alterations in gut microbiota at the phyla level, we performed a deeper analysis down to the species level. The richness of the gut microbiome is reflected as the total OTUs. As shown in figure 3, a marked difference in the baseline species-level bacterial profile between young and aged mice was identified. In addition, TBI resulted in further discordance between young and aged mice after injury. Based on the general-OTU dataset, age-linked and TBI-linked disease associated microbial networks (DAMNs) were identified (Table 1).

## Discussion

Age is well-recognized as an independent risk factor for poor outcome after TBI. The mechanisms underlying the effect of age, however, remain unclear. Age-related alterations in the gut microbiome are also becoming increasingly recognized as a contributing factor to a myriad of disease processes [31]. Our data revealed markedly different, yet stable, alterations in the gut microbiome due to age. Phylum and species level analysis revealed the sources of these age-related changes as well as differential, age-related, responses to TBI. Using the taxonomic classification of the OTUs we looked into multiple levels of taxonomy. Each level revealed a different part of the complex relationship between age, TBI, and the microbiome. First, there were no statistical differences in alpha diversity within samples of groups pre or post-TBI (data not shown) indicating no change in the total number of species expressed or lost secondary to TBI. We did find marked, age-related, differences in beta diversity indicating that differences in gut microbial composition may be bound to age (Figure 3). This analysis appears to confirm baseline differences physiology due to age, but at this level, none of the data corresponded with the differential neuropathology between age groups.

Probing the phyla and species taxa we found increased support for baseline physiological differences due to age and novel evidence of differential age-linked changes with injury. At the species level specifically, we were able to identify a separate group of age-dependent changes in dysbiosis within the fecal microbiome. Compared to young sham mice, each group (young TBI, aged sham, & aged TBI) showed a unique divergence in their bacterial profile. Cross-referencing with published data, the predominance of the shifts in bacterial abundance have been associated with diseases of the brain and gut [23]. The disease associated microbial network (DAMN) seen in aged sham mice was separate and distinct from those found in the young and aged TBI groups at the species level (Table 1). Differences in microbial profiles based on physiology is not novel. There are, however, many current efforts to target the microbiome as a therapy to ameliorate the various pathological effects of traumatic brain injuries could be informed by the finding of age-based variations in dysbiosis [25]. If these probiotic and fecal transplantation-based efforts are to be successful, the variation in bacterial networks according to age should be considered.

According to descriptions in the literature, the microbiomes of most healthy humans are dominated by the gram-positive Firmicutes and gram-negative Bacteroidetes [32]. This was consistent with the findings of our phyla level investigation. In our investigation, healthy young adult sham mice maintained a much higher level of expression of these phyla than aged mice at nearly every time point (Figure 2A). The outsized age-related variance pre-injury may help explain the observed differences in beta diversity. The phyla-level disparity between ages outweighed the phyla-level changes induced by injury. We also observed significant changes in Actinobacteria and Proteobacteria within young mice as compared to aged mice at the acute time points post-injury. Changes in these gram-positive phyla are linked to deficits in learning and memory and neurodegeneration [27,33].

We were able to most effectively characterize the interaction between age and TBI at the lowest taxa level (i.e., species) (Figure 3). At the pre-injury baseline, aged mice showed an increase in many species that have been previously linked with disease compared to young sham mice. While the presence of these species is not a direct harbinger, loss or overabundance of these species have been reported to play a key role in the persistence of inflammatory responses seen in chronic diseases of the brain and gut [34]. For example, our data show age-induced increases in Dorea (Multiple sclerosis), *Butyricicoccus pullicaecorum* (inflammatory bowel disease), *Allobaculum* (Alzheimer’s disease), *Candidatus* Arthromitus (systemic inflammation), *Streptococcus*, *Clostridium* (traumatic brain injury), Bacteroides (Alzheimer’s disease), *Parabacteriodes gordonii* (Inflammatory Bowel Disease)), *Prevotella* (systemic inflammation), and *Bacteroides ovatus* (metabolic disease and Alzheimer’s disease) [22,35-37]. On the other hand, the bacteria *Lactobacillus*, which is often used as a probiotic, and *Parabacteroides distasonis* have been linked to positive effects on anxiety and depression and have been shown to possibly alleviate obesity and metabolic disease in mice [38,39]. Both of these species were present in higher levels in aged mice in our study.

While post-TBI comparisons were admittedly highly influenced by the previously described age-induced variations, the expression of several microbial species were significantly altered in an age-dependent fashion post-TBI (Figure 3). Young TBI mice showed increased expression of several unfavorable species like *Clostridium methylpentosum* (systemic inflammation), *Allobaculum* (Alzheimer’s disease), *Clostridium clocleatum* (systemic inflammation), *Anaerostipes* (dysbiosis), *Lactobacillus*, *Turiobacter* (inflammatory bowel disease), and *Coprococus* (Parkinson’s disease). Similarly, decreases in other bacterial species correlated with inflammatory diseases were seen in the young TBI group compared to young shams. These included decreases in *Coprobaccillus* (inflammatory bowel disease and systemic inflammation), *Blautia* (Obesity), and *Dehalobacterium* (systemic inflammation) [37,40]. These variances were unique to the young mice post-injury and occurred in conjunction with unfavorable histologic outcomes in the young TBI mice [17]. On the other hand, aged mice demonstrated variance in a completely different set of microbial species after TBI. Aged TBI mice had increases in *Jeotgalicoccus psychrophilus* (uncharacterized) and *Lactobacilus reuteri* (intestinal probiotic) along with loss of *Candidatus Arthromitus* (probiotic, metabolic disease), *Bacteroides* (metabolic disease and Alzheimer’s disease), and *Anaerostipes* (metabolic disease) over time.

In a recent analysis, Treangen and colleagues also found temporally-linked divergences in baseline bacterial species. which differentiated after TBI at hyperacute 1-day time point. For example, after injury, they found that TBI-associated decreases in the beneficial strains of *L. gasseri* (metabolic disease), *Ruminococcus flavieciens* (amyotrophic lateral sclerosis), and *Lactobacillus* along with and increases in *E. ventriosum* (Obesity) and *M. formatexigens* (Probiotic, metabolic disease) [22]. Combined with our current study during the recovery phase of TBI, this points to a previously uncharacterized interaction between age, injury, and gut dysbiosis. While some reviews have suggested the use of probiotics to normalize the gut microbiome after injury and to treat subsequent disease, direct study is necessary to prove a benefit. The current study suggests that a more thorough characterization is required as native probiotic-associated bacteria are altered differentially both after injury, and with age. Future studies verifying the possible benefits of probiotic treatment post-injury may necessitate stratification of probiotic strains by age. The generous addition of even beneficial bacteria could lead to further dysbiosis and pathology [25,37,40-42]. The limitations of the present study merit consideration. First is the utilization of the CCI model which delivers a focal injury with limited diffuse effects. CCI allows for tight control of injury parameters and consistent reproducibility between experiments. However, the focused nature of the impact may not fully recapitulate the disparate nature of TBI in human patients. Further, analysis of neurological status, motor function, cognitive status, frontal lobe function, and the functional outcomes of microbial changes would provide more mechanistic insight. Additionally, we focused on the influence of age in mice with similar brain injuries. We were required to exclude female mice from this study due to significant size differences that would impact the depth and scope of the TBI. Multiple researchers have outlined sex differences in TBI [43-45]. In order to control the level of variability we therefore decided to focus on sex-based differences in TBI in a separate study currently underway.

While the current study shows an age-related effect of fecal dysbiosis after severe TBI, further study is needed to identify mechanisms linking the gut microbiome profile with and the direction, progression, and outcome of traumatic brain injury. There are previous reports of chronic bidirectional brain-microbiome interactions after TBI as well as differential outcomes related to age [16,46-48]. The current work, however, is the first to characterize the interaction of age on the microbiome over the course of TBI. These data suggest a divergent pathophysiology of injury between young and aged groups reflecting a previously unknown interaction between age, TBI, and the gut-brain axis. Alterations in the gut microbiome have been previously characterized in a number of neurologic and neurodegenerative disorders [49]. In fact, gut dysbiosis is heavily implicated in the susceptibility, acceleration and exacerbation of cerebrovascular disease, Alzheimer’s disease, and Parkinson’s disease [50-52]. Indeed, restoring a healthy gut microbiome has shown some benefit slowing the progression of these disease processes [53-55]. Although the relationship between TBI and the gut microbiome is still relatively unknown, the data from these other neurologic and neurodegenerative processes gives hope to the possibility that restoration of pre-injury gut microbiota may represent a novel therapeutic approach to this highly morbid injury process.

## Conclusion

There were marked, age-related, differences in beta diversity between young and aged mice indicating that differences in gut microbial composition may be bound to age. Taken together with the previous study identifying age-dependent neuropathological and functional changes this work highlights. The past decade has seen a paradigm shift in our understanding of the brain-gut axis. Studies have outlined important interactions between the intestinal microbiota and the brain. In identifying these biological mechanisms, clinical and preclinical work has identified novel targets for the potential treatment of neurologic disorders, including Alzheimer’s disease, autism spectrum disorders, anxiety, depression, and others. Herein we present evidence of a distinct and complex pathologic phenotypes in TBI based on aged. Using this evidence, we assert that similar study and modulation of microbial phyla or species could reveal viable treatments that have remained elusive in the arena of TBI. A robust analysis that accounts for the interplay of influential variables such as age, microbial makeup, or sex could yield better results within groups. Our data implicates the interplay of age, injury, and the gut microbiome in differential outcomes after TBI pointing towards a new area of potential therapy for this highly morbid injury process.

## Supplementary Material

JEN-21-044_Supplementary file

## Figures and Tables

**Table 1:** Disparate networks of disease associated microbe revealed with age and injury. The predominance of bacterial shifts seen in the heatmap (Figure 3) have published links to pathology of the brain-gut axis. Listed here are the names of bacterial species that were altered in mice with age and injury. The data is presented in conjunction with pathological processes associated with similar changes seen in the literature. Contrast of aged shams with young sham revealed aged mice to have the largest shift of bacterial species. Comparison with of young and aged injured animals to their respective sham groups reveal the dissimilar yet pernicious expression of bacterial species of linked to BGA dysfunction.

Disease associated microbial network in aged sham	BGA Associated Disease	Disease associated microbial network in young TBI	BGA Associated Disease	Disease associated microbial network in aged TBI	BGA Associated Disease
Dorea	Inflammatory Bowel Syndrome(IBS),Mult iple Sclerosis(MS)	Methylpentosum	SI	Jeotgalicoccus psychrophilus	Uncharacterized species
Butyricicoccus pullicaecorum	Ulcerative Colitis (UC) and Crohn's Disease (CD)	Allobaculum	AD	Lactobacilus reuteri	Metabolic Probiotic (MP)
Allobaculumx	Alzheimer’s Disease (AD)	Clostridium clocleatum	SI		
Candidatus Arthromitus	Systemic Inflammation (SI)	Anaerostipes	Gut Dysbiosis (GD)		
Streptococus, Clostridium	Traumatic Brain Injury (TBI)	Lactobacillus, Turiobbacter	IBD		
Bacteroidetes	Metabolic Disease (MD), (AD)	Coprococus	Parkinson’s Disease (PD)		
Bacteroides	MD, AD				
Parabacteriodetes gordonii	Inflammatory Bowel Disease (IBD), UC				
Prevotella	SI				
Bacteroides ovatus	MD, AD				
					
Underrepresented Microbial Species		Underrepresented Microbial Species		Underrepresented Microbial Species	
Staphylococcus succinus	Attenuates Inflammation			Candidatus arthromitus	MP
Clostridium	Attenuates depression, Enteritis			Bacteroidetes	MP, AD
Clostridium Cocleatum	Colitis			Anaerostipes	MD

## Data Availability

The data that support the findings in this manuscript are available from the corresponding author upon request. The 16S rRNA gene data have been deposited to the NCBI BioProject data repository with the following dataset identifier: “BioProject ID: PRJNA722465” with the title “Age Alters Dysbiosis and Neuropathology In Traumatic Brain Injury”.
