# Supplementary material for: Differential Fecal Microbiome Dysbiosis after Equivalent Traumatic Brain Injury in Aged Versus Young Adult Mice
Source: J Exp Neurol. Author manuscript; Available in PMC 2021 Nov 24. (PMC8612634; doi:10.33696/neurol.2.044)
Supplement: JEN-21-044_Supplementary file [file NIHMS1745139-supplement-JEN-21-044_Supplementary_file.pdf]

Supplemental Data:

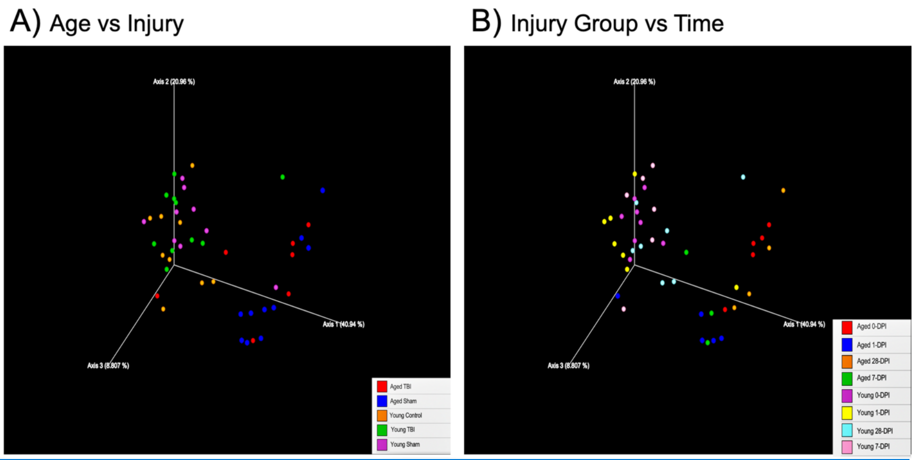

Supplemental Figure 1. Fecal bacterial microbiome beta diversity in young and aged mice. *Principal Coordinates Analysis (PCoA) plot reveals no statistically significant differences in beta diversity due to time or injury. The PCOA represents all subjects at every timepoint based on 16S rRNA amplicon sequencing (N=18). Each colored dot represents an individual mouse at a different day post injury (DPI) with directionality and association indicating the bacterial profiles similarity.*
